# Characterization of Fractal Structures by Spray Flame Synthesis Using X-ray Scattering

**DOI:** 10.3390/ma15062124

**Published:** 2022-03-14

**Authors:** Mira Simmler, Manuel Meier, Hermann Nirschl

**Affiliations:** Institute of Mechanical Process Engineering and Mechanics, Karlsruhe Institute of Technology, 76131 Karlsruhe, Germany; manuel-meier@outlook.com (M.M.); hermann.nirschl@kit.edu (H.N.)

**Keywords:** small-angle X-ray scattering (SAXS), nanoparticle characterization, fractal structures, spray flame synthesis (SFS), flame spray pyrolysis (FSP), metal oxides, zirconia

## Abstract

In this work, we take on an in-depth characterization of the complex particle structures made by spray flame synthesis. Because of the resulting hierarchical aggregates, very few measurement techniques are available to analyze their primary particle and fractal properties. Therefore, we use small-angle X-ray scattering (SAXS) and transmission electron microscopy (TEM) to investigate the influence of the precursor concentration on the fractal structures of zirconia nanoparticles. The combination of information gained from these measurement results leads to a detailed description of the particle system, including the polydispersity and size distribution of the primary particles. Based on our findings, unstable process conditions could be identified at low precursor concentrations resulting in the broadest size distribution of primary particles with rough surfaces. Higher precursor concentrations lead to reproducible primary particle sizes almost independent of the initial precursor concentration. Regarding the fractal properties, the typical shape of aggregates for aerosols is present for the investigated range of precursor concentrations. In conclusion, the consistent results for SAXS and TEM show a conclusive characterization of a complex particle system, allowing for the identification of the underlying particle formation mechanism.

## 1. Introduction

The production of metal oxide nanoparticles has gained importance in recent decades because of their specific application properties. Their small size leads to a high surface-to-volume ratio and other effects, such as high porosity, a lower melting point and high van-der-Waal forces [1,2]. Depending on the composition, metal oxides can show para magnetism (iron oxides) [3], photocatalytic behavior (titania) [4], high toughness and wear resistance (zirconia) [5], electrical conductivity (aluminum-doped zinc oxid) [6] or insulation properties (silica) [7]. One approach to produce metal oxide nanoparticles, is flame spray synthesis, which is generally a fast process, very flexible in terms of switching material systems and relatively easily scaled up to industrial production rates [8,9]. Historically, this process is called flame spray pyrolysis (FSP) because of the production of carbon black in fuel-rich flames. For the production of metal oxides, the term spray flame synthesis (SFS) is more fitting, as an oxygen-rich flame is needed to prevent the formation of soot. A liquid precursor containing the metal ions is atomized and ignited. Depending on the precursor solution, a continuous flame is necessary to keep the turbulent spray flame burning [2,8,10]. The particle structure of the final product is a result of the underlying formation and growth mechanisms. The particle formation starts with a droplet leading to different particle systems depending on the flame conditions and precursor composition. The so-called gas-to-particle route produces aggregates consisting of very fine primary particles with a high specific surface area. The fine spray droplets turn to precursor vapor, forming molecules and clusters. They nucleate, coagulate and sinter to small nanoparticles, composing agglomerates and then aggregates. Other kinds of structures are solid particles in the micrometer range occurring by precipitation in the droplet and subsequent aggregation and sintering. These particles turn into hollow spheres or fragments of shells if the precipitation takes place on the surface of the droplet [4,11]. Because of the liquid feed, almost every element of the periodic table can be turned into an oxide, as the precursor only needs to dissolve in a liquid to form a sprayable solution [10]. The results presented in this work were obtained with the standardized burner *spraysyn* of the priority program “Nanoparticle Synthesis in Spray Flames” of the German Research Foundation (DFG) [2,12]. The goal is the reproducible production of fractal aggregates consistent in shape and size. However, the characterization of aggregates represents a challenge, as the particle system in question has multiple structural levels: crystals or clusters form primary particles, which in turn form aggregates. Crystal properties can be analyzed with common measuring techniques, such as as X-ray diffraction (XRD) or wide-angle X-ray scattering (WAXS), using the Scherrer equation for the Bragg peaks [13]. The investigation of particle properties is more difficult. Measurement techniques such as dynamic light scattering (DLS) for suspensions or scanning mobility particle sizing (SMPS) for aerosols struggle with non-spherical and polydisperse particle systems [10]. One way to gain information about the primary particle size of polydisperse systems is the determination of the specific surface area. Here, gas adsorption using the Brunauer–Emmett–Teller (BET) theory allows for the calculations of an average size for spherical particles [14]. Transmission electron microscopy (TEM) can determine particle distributions and the state of sintering between the primary particles [15]. The shape and morphological features of aggregates can be described with the concepts of fractal dimension [16,17]. However, only measurement techniques based on electron microscopy or light/X-ray scattering provide the necessary information [17]. Here, wide-angle light scattering (WALS) has shown great potential to analyze the shape and size of aggregates in the micrometer range [18].

Similar to WALS but on the lower nanoscale, small-angle X-ray scattering (SAXS) combines significant particle characteristics such as fractal properties and crystal, primary particle and aggregate size in one data set. Although the sample amount here is smaller than for BET, SAXS is an integrative measurement method providing representative data. For complex particle systems, the overlapping scattering information has to be separated to extract accurate results for each particle characteristic. It is possible to derive a particle size distribution or calculate a polydispersity index. Research proves that this is a valuable technique for nanoparticles, generally and specifically primary particles made by flame synthesis [15,19,20].

The aim of this paper is the characterization of fractal structures of zirconia made by SFS using SAXS and TEM. Specifically, we investigate the influence of the precursor concentration in SFS on primary particle and fractal properties of the final product. Based on the scattering data, different methods to derive an average diameter for the primary particles are discussed and compared with diameters obtained from TEM. In a further step, we analyzed the polydispersity of the primary particles leading to first insights of the process conditions. Furthermore, volume size distributions of the primary particles are calculated from SAXS and TEM data and associated with fractal information of the whole particle system gained from SAXS scattering. Finally, using the combined results of the in-depth SAXS and TEM characterization obtained in this work, the particle formation mechanism leading to the presented fractal structures can be identified from existing models for SFS.

## 2. Materials and Methods

### 2.1. Particle Synthesis and Characzterization

The zirconia particles were produced in a spray flame with the *spraysyn* burner (University of Duisburg-Essen, Duisburg, Germany) using mass flow controllers by Bronkhorst (Karmen, Germany). The laminar pilot flame stems from 2 slm (standard liters per minute) methane and 16 slm oxygen and is stabilized with 120 slm sheath air (pressured air). A syringe pump (Harvard Instruments, Holliston, MA, USA) pumps 2 mL min^−1^ of liquid precursor through the cannula into the center of the burner, which is atomized with 10 slm oxygen through an annular gap around the cannula. The pilot flame ignites this spray to generate a spray flame. The exact dimensions of the burner can be found in Schneider et al. [12].

The precursor solution consisted of butanol (1-Butanol, 99.5% for analysis Acros Organics by Thermo Fisher Scientific, Waltham, MA, USA) and varies concentration *c* on zirconium butoxide from *c* = 0.05 mol L^−1^ to *c* = 1.0 mol L^−1^. Zirconium butoxide comes as an 80% solution in butanol (Zirconium(IV)-n-butoxide 80%(*w/w*) in butanol, Alfa Aesar by Thermo Fisher Scientific, Waltham, MA, USA).

The nanoparticles were extracted with a hole-in-tube probe similar to Tischendorf et al. [15] at 120 mm above the burner surface. The tube consists of stainless steel for high temperatures with an outer and inner diameter of 10 and 8 mm, respectively. The diameter of the hole measures 0.7 mm with a thickness of wall of 0.5 mm. The tube connects to a filter with a track etch membrane (Whatman Nuclepore Track-Etched Membranes, Merck, Darmstadt, Germany) with pore structures of 200 nm. A venturi jet (ESSKA, Hamburg, Germany) provides a necessary vacuum, which is reduced by an air flow of 10 slm in the tube.

The powder samples from the membrane were analyzed using the transmission electron microscope JEOL JEM-2200FS at the Interdisciplinary Center for Analytics at the Nanoscale (ICAN) at the University Duisburg Essen (Duisburg, Germany). To calculate an averaged Feret diameter, over 500 particles were considered.

For further particle characterization, the SAXS laboratory camera Xeuss 2.0 Q-Xoom (Xenocs SA, Grenoble, France) was used at the Institute of Mechanical Process Engineering and Mechanics at the KIT (Karlsruhe, Germany). The camera is equipped with the X-ray micro focus source Genix3D Cu ULC (Ultra Low divergence) of Cu-k-alpha with an energy of 8.04 keV and a wavelength of 1.5406 Å. The collected powder was transferred to a polyimide foil and measured at a sample-to-detector distance of 1750 mm and an exposure time of 10 min without a beam stop using the Pilatus3 R 300K detector (Dectris Ltd., Baden, Switzerland). After azimuthal integration and background correction, the data were converted to absolute scaling using standardized glassy carbon.

### 2.2. Method of Small-Angle X-ray Scattering (SAXS)

To compare different SAXS devices and setups the scattering vector *q* is used consisting of the wavelength *λ* and the scattering angle *θ* and is defined as follows:

(1) $$q=\frac{4\pi }{\lambda }\;\;\sin\left(\frac{\theta }{2}\right)$$

The scattering data are typically presented in a double logarithmic diagram with the intensity *I* in cm^−1^ on the *y*-axis and the scattering vector *q* in Å^−1^ on the *x*-axis. The Unified Fit Model (Irena Package 2.68 [21], IgorPro, WaveMetrics Inc., Portland, OR, USA) according to Beaucage [22] was used to evaluate the collected scattering data by dividing it into different structural levels. Each level is assigned a local Guinier fit and a local power law fit. The local Guinier fit indicates the averaged characteristic size of the particles at a specific structure level *i* using the radius of gyration *R_gi_* and prefactor *G_i_* [13,23].

(2) $$I\left(q\right)=G_{i}\exp\left(-\frac{q^{2}\;R_{gi}{}^{2}}{3}\right)$$

The local power law fit provides information about the morphology of the underling structure level *i*, where the exponent *p_i_* indicates the slope and the prefactor *B_i_* the intercept with the *y*-axis [13,24,25].

(3) $$I\left(q\right)=B_{i}\;q^{-p_{i}}\;$$

**Figure 1:**
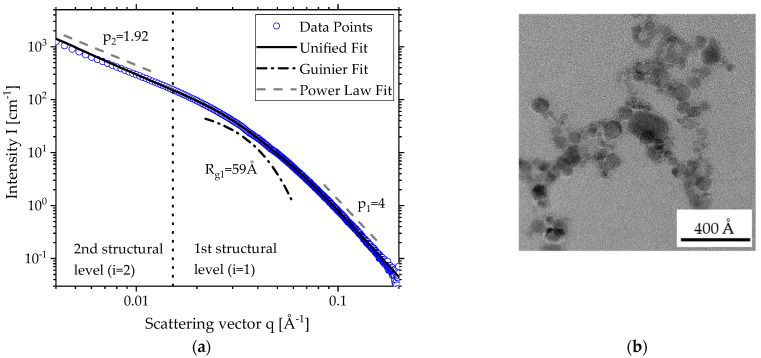
(**a**) SAXS scattering data and local fits for zirconia made by SFS with *c* = 0.4 mol L^−^^1^; (**b**) TEM image for zirconia made by SFS with *c* = 0.4 mol L^−1^.

As the TEM image in Figure 1b depicts, particles produced by SFS typically have two structural levels where the small primary particles form the 1st (*i* = 1) level and the consequentially resulting aggregates the 2nd (*i* = 2) level. Figure 1a shows the scattering curve (blue dots) for zirconia produced from a precursor of *c* = 0.4 mol L^−1^ zirconium butoxide in butanol with its two structural levels. The solid black line depicts the Unified Fit [22] with the corresponding local Guinier fit (dashed dotted line) for the primary particles in *i* = 1 and two local power law fits for both levels *i* = 1, 2 (dashed line) above and under the fit for better legibility. The errors of the scattering were omitted because they range in the size of the data points. To demonstrate that zirconia was formed during the spray flame synthesis (SFS), Figure S1 in the Supplementary Materials shows WAXS (wide-angle X-ray scattering) data of zirconia produced with a precursor concentration *c* = 0.4 mol L^−1^ [26,27,28].

For *i* = 1 describing the primary particles, the Guinier fit is positioned at the change of slope at about *q* = 0.015 Å^−1^. For the particle size *R_g1_* = 59, Å was determined.

For *i* = 2, the aggregate size exceeded the limit of resolution of SAXS. To determine a radius of Gyration, a module for ultra-small-angle X-ray scattering (USAXS) is needed.

The gradual change of slope in Figure 1a indicates a high polydispersity of the primary particles [14]. It can be described by the geometric standard deviation *σ_g_*, which is just the exponential function of the polydispersity index (*PDI*) using the parameters of both the local Guinier and the power law fit:

(4) $$\sigma_{g}=\;\exp\left(\sqrt{\frac{\ln\left(PDI\right)}{12}}\right);\;\;\;\;\;\;\;PDI=\frac{B_{1}\;R_{g1}{}^{4}}{1.62\;G_{1}}$$

To compare *R_g_*_1_ with primary particle sizes of other measurement techniques, a mean diameter based on spherical particles can be calculated based on *R_g_*_1_ and the *PDI* [29]:

(5) $$d_{R_{g1}}=2\sqrt{\frac{5}{3}}R_{g1}\exp\left(-13\frac{\ln\left(PDI\right)}{24}\right)$$

Another way to calculate a particle diameter is using the specific surface area (*SSA*) and the density *ρ* of the material defined as [14]

(6) $$d_{SSA}=\frac{6\;Q}{\pi \;B_{1}}=\frac{6}{\rho \;SSA}$$

with *B*_1_ and *Q* as Porod Invariant.

The Porod Invariant *Q* is defined as the area under the scattering data in the Kratky Plot (*I* * *q*^2^ over *q*):

(7) $$Q=\int_{0}^{\infty }q^{2}\;I\left(q\right)\;dq$$

To calculate *d_SSA_* for the primary particles in aggregates, only the 1st structural level (*i* = 1) should be used for the limits of the integral of *Q* [14].

To gain further information about the particle size, the Model Size Distribution (IgorPro, WaveMetrics Inc., Portland, OR, USA) can be applied to the scattering data [30]. The model splits the analyzed section of the scattering curve into a fixed number of intervals for the size distribution and compares the calculated scattering curves for assumed size distributions with the measured scattering data. The mathematical solution for this problem is based on the maximum entropy method [31,32].

Information about the fractal properties of the particle system can be gained from the power law fits. If the slope p_1_ of the 1st structural level is four (see Figure 1a), the fit is called Porod fit and the particles show Porod behavior, meaning a sharp interface [13]. This is typically for crystalline nanoparticles and also the case for the power law fit of the 1st structural level in Figure 1a. If the slope is lower, the surfaces are rougher. However, a value of three marks the physical limit.

For the 2nd structural level, the exponent *p_2_* (see Figure 1a) represents the fractal dimension of mass for the aggregates and gives information about their shape [16]. A value of one suggests stringy aggregates, whereas two suggests a sheet and three suggests structures in all geometrical dimensions [13,17]. The derived value of 1.54 in Figure 1a is lower than typically expected for flame-made products. Following the diffusion-limited cluster aggregation, clusters stick to other clusters when meeting them through random motion to form aggregates. This means that the diffusive motion of the clusters is the limiting step in this process and leads to fractal dimensions of 1.7–1.8 [33,34]. An electrical field in the flame could possibly lead to an orientation of the aggregates and subsequently a reduction in dimensionality [35,36].

## 3. Results and Discussion

### 3.1. Radius of Gyration and Primary Particle Diameter

**Figure 2:**
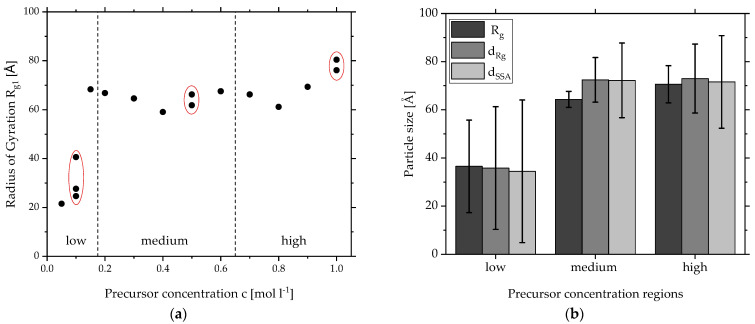
(**a**) Radii of gyration from SAXS data of primary particle level made by SFS from different precursor concentrations grouped in regions: low (*c* = 0.0–0.15 mol L^−1^), medium (*c* = 0.2–0.6 mol L^−1^) and high (*c* = 0.7–1.0 mol L^−1^). (**b**) Averaged primary particle size for each region of precursor concentration calculated from SAXS data: radius of gyration, diameter based on radius of gyration, diameter based on specific surface area.

To gain insights into the influence of the precursor concentration on the resulting nanoparticle structure, SFS experiments with different precursor concentrations of zirconium butoxide were conducted with the *spraysyn* burner under constant flame conditions. As explained above, Figure 1a shows the typical scattering data obtained from the collected powder with a clear local Guinier Region for the primary particle level *i* = 1. To compare the scattering data from different precursor concentrations, Figure 2a shows the determined *R_g1_* by the local Guinier fit Equation (2) for each concentration ranging from *c* = 0.05 to 1.0 mol L^−1^. Multiple experiments are grouped with a red ellipse. For *c* > 0.2 mol L^−1^, the *R_g1_* stays around 60–80 Å. Second experiments with *c* = 0.5 mol L^−1^ and *c* = 1.0 mol L^−1^ yield similar results, indicating a stable and reproducible process. It seems that an increase in precursor concentration does not lead to a larger primary particle size but only to a higher particle number. For *c* < 0.2 mol L^−1^, the *R_g_*_1_ increases from 20 Å to 70 Å. At first glance, this seems like an indication for growth. However, there are three different results of *R_g_*_1_ for *c* = 0.1 mol L^−1^ ranging from 20 to 40 Å, implying instable process conditions.

To be able to compare these results with other measurement techniques, the radius of Gyration needs to be converted into a geometric diameter. Equations (5) and (6) use different parameters of the Guinier and Porod Fit to calculate a geometric diameter equivalent to a sphere. The *R_g_*_1_ is the basis for Equation (5), whereas Equation (6) is independent of this parameter and is based on the SSA instead. Both diameters were calculated for every precursor concentration shown in Figure 2a. For a clearer presentation, the precursor concentration spectrum of this work is divided into three regions: low (*c* = 0.0–0.15 mol L^−1^), medium (*c* = 0.2–0.6 mol L^−1^) and high (*c* = 0.7–1.0 mol L^−1^).

The results in each region were averaged and a standard deviation was calculated. Figure 2b compares the averaged results in each region for the radius *R_g_*_1_, the diameter *d_Rg_* using Equation (5) and the diameter *d_SSA_* using Equation (6) in a bar chart. Focusing on the average values, all results in one region are very similar. Both ways to calculate a diameter from SAXS fits yield almost the same results, showing good accuracy of the Guinier and Porod fit. The *R_g_*_1_ is also in the same range as the geometric diameters. This is a sign for high polydispersity, as bigger particles are overrepresented by intensity measurements compared to smaller ones. This leads to a higher value for the *R_g_*_1_ [14]. However, both calculations of the diameter take this into account: in Equation (5), the polydispersity index (*PDI*); in Equation (6), the *SSA*. In absolute numbers, this means an averaged smaller particle size of around 35 Å for the lower precursor concentrations (*c* < 0.2 mol L^−1^) and a consistent larger size of around 70 Å for medium and high concentrations (*c* = 0.2–1.0 mol L^−1^).

Having a look at the error bars, which represent the standard deviation of the averaged results, they are significantly higher for the low-concentration region. This is expected considering the results of Figure 2a for concentrations lower than *c* = 0.2 mol L^−1^ and strengthens the claim of instable process conditions. The difference between the error bars for the medium and high concentrations are noticeable but they are not significantly higher for the higher concentrations. Focusing now on the deviations in one concentration region, a trend is visible for all three regions: *R_g_*_1_ shows the lowest deviation; *d_SSA_* shows the highest deviation; *d_Rg_*_1_ is in the middle.

**Figure 3:**
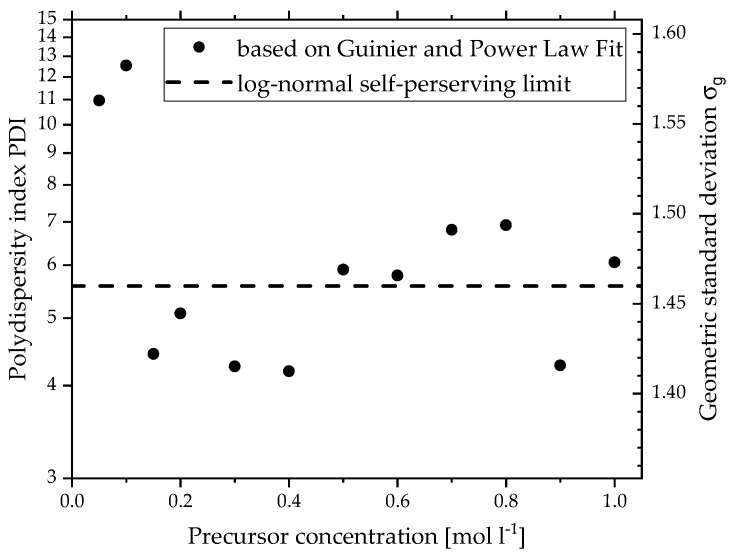
Polydispersity index and geometric standard deviation calculated from SAXS data using Equation (4) of primary particles made by SFS from different precursor concentrations.

Since these deviations are just a result of the average in one concentration region, it does not describe the polydispersity of the primary particles of one precursor concentration. As mentioned before, the slow change of slope of the SAXS curve in Figure 1a indicates a high polydispersity for this particle system, which the Guinier fit cannot model. One tool to express this property is the *PDI* by Beaucage based on *B*_1_, *G*_1_ and *R_g_*_1_^4^ (Equation (4)). The *PDI* is also part of Equation (5) and can be directly converted to the geometric standard deviation *σ_g_.* Figure 3 depicts the *PDI* on the left and *σ_g_* on the right *y*-axis for every precursor concentration (*x*-axis). The values are closely scattered around the self-preserving limit for aerosol growth of *σ_g_* = 1.46 or *PDI* = 5.56 for the free-molecular regime of particle transport by Friedlander [37] (dotted line) and are in agreement with results from similar experiments [14,29,38]. This indicates stable process conditions and that a high polydispersity is typical for flame-made particles. For the lowest precursor concentrations, however, the *PDI* shows very high values, around double the self-preserving limit, which cannot be connected to any model. This supports the earlier claim of very high polydispersity due to unstable process conditions. Overall, the SAXS evaluation of the primary particle size reveals a stable diameter of 70 Å and a typical *PDI* for aerosol for *c* > 0.2 mol L^−1^, whereas low precursor concentrations lead to smaller particle diameters and very high polydispersity.

### 3.2. Comparison of SAXS and TEM Results

#### 3.2.1. Mean Diameter

**Figure 4:**
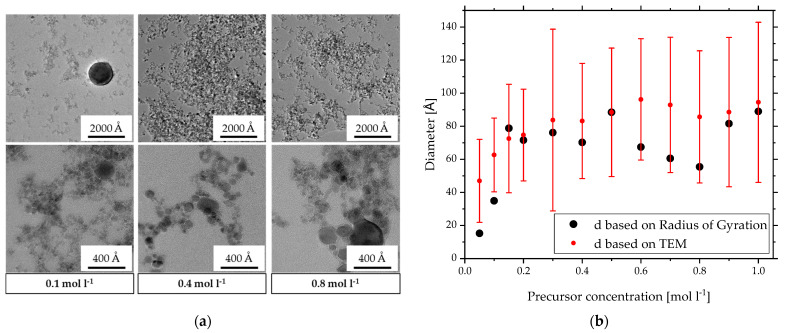
(**a**) TEM images in two resolutions of zirconia nanoparticles made by SFS with three different precursor concentrations. (**b**) Comparison of diameters based on TEM and SAXS of primary particles made by SFS with different precursor concentrations.

To confirm these results of SAXS with another measurement technique, a detailed TEM analysis was performed to compare mean diameters and the size distribution of the primary particles. Figure 4a shows representative TEM images of the product of three different precursor concentrations in two resolutions. The lower resolution gives a broad overview of the particle system depicting small nanoparticles in fractal structures. The primary particles of *c* = 0.4 mol L^−1^ and *c* = 0.8 mol L^−1^ have a higher contrast and seem slightly bigger than the ones of *c* = 0.1 mol L^−1^. One very big particle of over 1000 Å is also visible in the image of *c* = 0.1 mol L^−1^ precursor concentration. Having a closer look, the higher resolution shows a broad polydispersity in size of the primary particles for all three precursor concentrations. Here, *c* = 0.1 mol L^−1^ also has the lowest contrast and the smallest sizes lower than 50 Å with one particle of around 200 Å. In comparison, *c* = 0.4 mol L^−1^ shows a smaller distribution with particles roughly between 80 and 200 Å. For *c* = 0.8 mol L^−1^, the particle distribution seems wider and ranges up to 350 Å. This takes only in what is visible in these few images but confirms the results so far: for lower concentrations, a high polydispersity is evident with single larger particles. This can explain the differences in the radii of gyration of the three experiments with *c* = 0.1 mol L^−1^ in Figure 2a. Since intensity measurement techniques are very sensitive to size, even a few larger particles can influence the scattering results vastly. So, if the number or the size of these larger particles varies slightly in the SAXS sample, it effects the results significantly. Although the other two precursor concentrations also resulted in polydisperse primary particles, no single large particles of 1000 Å could be observed. This is represented in the stable radius of gyration in Figure 2a and the *PDI* in Figure 3. The TEM images even confirm the low *PDI* of 4.2 for *c* = 0.4 mol L^−1^ compared to the medium *PDI* of 6.9 for *c* = 0.8 mol L^−1^.

Figure 4b compares the calculated average diameter *d_TEM_* of these particles and the resulting standard deviation (red) with *d_Rg_* calculated with Equation (5) (black). Overall, the TEM data show the same trends as SAXS with smaller particle sizes starting at 35 Å for *c* < 0.2 mol L^−1^ and a constant diameter between 70 and 90 Å for higher concentrations. The standard deviation of *d_TEM_* illustrates the polydispersity among the primary particles and makes differences in averaged size for *c* > 0.2 mol L^−1^ negligible. Almost for every sample, *d_TEM_* overestimates *d_Rg_* but includes it in the deviation. This shows good agreement between SAXS and TEM data and, therefore, supports the assumption of the adjustment from an intensity-based radius of gyration to a number based spherical diameter in Equation (5). It almost seems like an overcompensation due to lower values than the averaged diameter based on TEM. However, compared to SAXS measurements, the investigated number of particles in TEM is, with 500, significantly lower, which can cause some discrepancy. Additionally, these particles were picked by hand with a possible bias for higher contrast and therefore statistically larger particles. This especially seems to be the case for the lower precursor concentrations resulting in the smallest particle sizes, as is the case in Figure 4a in the TEM image of lower resolution for *c* = 0.1 mol L^−1^. Considering the high standard deviation, the similarity between the mean primary particle diameter derived of TEM and SAXS is intriguing and demands further investigation.

#### 3.2.2. Size Distribution

**Figure 5:**
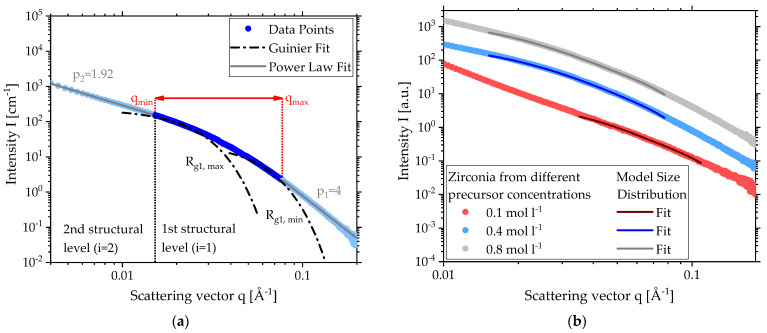
(**a**) SAXS scattering data and local fits to define the range of scattering vectors for the size distribution fit for zirconia made by SFS with *c* = 0.4 mol L^−1^. (**b**) SAXS scattering data for zirconia made by SFS with different precursor concentrations and the fits of the Model Size Distribution (*c* = 0.1 mol L^−1^, *c* = 0.4 mol L^−1^, *c* = 0.8 mol L^−1^).

A size distribution can reveal information about a standard deviation or *PDI* such as a bimodality. Therefore, size distributions of the primary particles were determined for one concentration in each defined region of precursor concentration using the Model Size Distribution (IgorPro, WaveMetrics Inc., Portland, OR, USA) on the corresponding SAXS data sets. This modeling tool is designed for single particles, not for aggregates. Since no adequate tools for fractal particle systems exist, the Model Size Distribution is a start to describing the high polydispersity in the primary particles in more detail. Therefore, the fitting range of the SAXS data has to be considered before applying the model. Figure 5a shows the scattering data (blue dots) for zirconia of *c* = 0.4 mol L^−1^ with its two structural levels and the local power law fits (gray lines). Instead of the mean Guinier fit for the 1st level given in Figure 1a, Guinier fits representing the minimum and maximum radius of gyration *R_g_*_1,*min*_ and *R_g_*_1,*max*_ were applied to define the range of scattering vectors for the size distribution fit (red arrow).

Because the program specification is based on single particle analysis, the lower limit of the scattering vector for the size distribution *q_min_* was set to the transition of the power law fit of the 2nd structural level to the Guinier fit of the 1st structural level. In contrast, SAXS data of single particles will lead to slope *p* = 0 for smaller scattering vectors just like the Guinier fit. For aggregates formed by primary particles, the slope *p* in this region can assume values between 1 and 3 according to the shape of the aggregate known as the fractal dimension of mass (see end of Section 2.2). This means that no larger particles, which would scatter in the region of the 2nd structural level, are considered for the size distribution.

The other end of the range *q_max_* is defined as the transition point of the 1st Guinier fit and the 1st power law fit. If the region of the 1st power law fit is considered for the size distribution fit, additional smaller particle fractions will appear in the size distribution. This is not feasible as the limit of the Guinier region *R_g_*_1,*min*_ is clearly reached and only the Porod region with *p* = 4 remains.

Figure 5b provides the SAXS data (lighter colored dots) and the calculated fits (darker colored lines) using the Model Size Distribution for a low (*c* = 0.1 mol L^−1^), a medium (*c* = 0.4 mol L^−1^) and a high (*c* = 0.8 mol L^−^^1^) concentration. To display all three data sets in one diagram, the SAXS data were shifted in intensity to avoid overlapping. As described in Figure 5a, the region of the fit depends on the range of scattering vectors defined by *R_g1,min_* and *R_g1,max_* to include only the scattering of primary particles for the size distribution. In contrast to *c* = 0.4 mol L^−1^ and *c* = 0.8 mol L^−1^, the sample of *c* = 0.1 mol L^−1^ with *p* = 3.5 does not show Porod behavior but the same principle was still applied using the 1st power law fit. The exact values for range of scattering vectors for the size distribution fit as well as the slope *p_1_* of the 1st power law fit can be found in Table 1.

**Figure 6:**
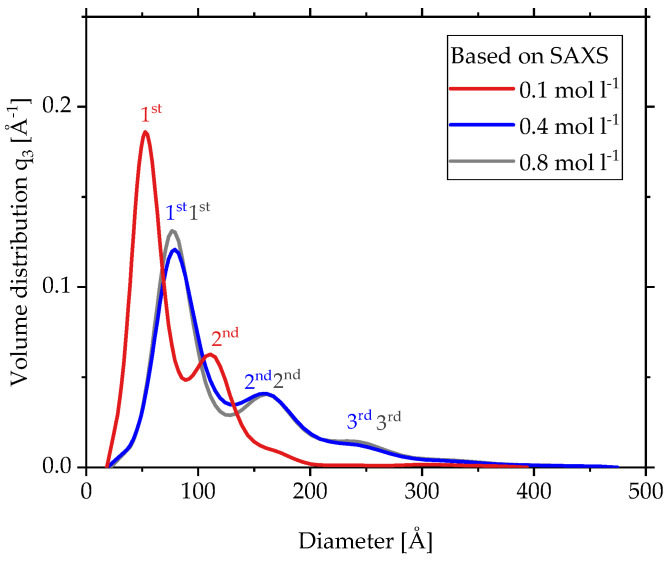
Volume size distribution with numbered peak calculated from SAXS data of primary particles made by SFS with three different precursor concentrations.

The fits of Figure 5b lead to the calculated volume size distributions of the 1st structural level in Figure 6 with numbered peaks for the same three concentrations(*c* = 0.1 mol L^−1^, *c* = 0.4 mol L^−^^1^, *c* = 0.8 mol L^−1^). Each distribution shows multimodal behavior with a significantly higher peak for the smallest fraction. With increasing precursor concentration from *c* = 0.1 mol L^−1^ to *c* = 0.4 mol L^−1^, the distributions shift to larger particle sizes, while from *c* = 0.4 mol L^−1^ to *c* = 0.8 mol L^−1^ the size distribution does not change anymore. This behavior is in good agreement with the results so far. The exact diameters of the peaks are given in Table 2. The position of the 1st peak grows from 55 to 80 Å with increasing precursor concentration and then stays there for high concentrations. Although these diameters are volume based, the SAXS and TEM diameters based on number in Figure 4b show very similar values. The position of the 2nd peak for each concentration is roughly double the diameter of the 1st peak, and together the 2nd and 3rd peak clearly illustrate the existence of larger particles independent of the concentration.

**Figure 7:**
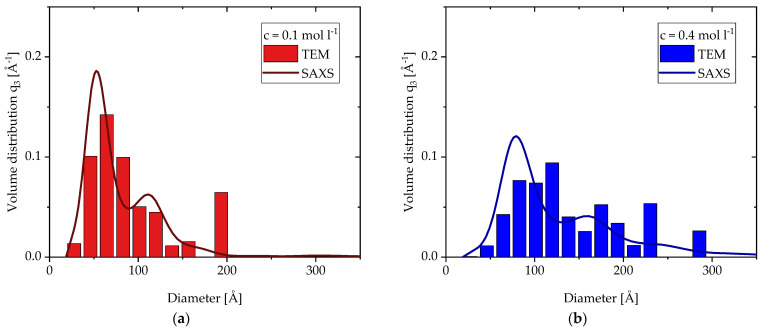
Volume size distribution calculated from TEM and SAXS data of primary particles made by SFS (**a**) with a precursor concentration of *c* = 0.1 mol L^−1^ and (**b**) with a precursor concentration of *c* = 0.4 mol L^−1^.

In addition to the SAXS results, the TEM analysis of the particles also allows for the calculation of a volume size distribution. Figure 7a and 7b show the comparisons of these distributions for *c* = 0.1 mol L^−1^ and *c* = 0.4 mol L^−1^, respectively. Since the TEM distributions are based on 500 particles, they look less smooth than the modeled distribution of the SAXS data. Nonetheless, they are in good agreement with the SAXS distributions and show similar trends: a broad distribution consisting of the smaller particle fraction and some larger particles resembling a multimodal distribution with a few outliers. The similarity in these results support the use of the Model Size Distribution in this case of aggregates with the discussed fitting ranges. Compared to the SAXS distributions, the 1st peak of the TEM distributions are broader and seem to shift slightly to larger diameters for both concentrations. This phenomenon was also observed for the averaged data in Figure 4b. In contrast to the fairly clear 1st peak, a 2nd peak cannot be identified. It seems to be more a collection of outliners due to their low numbers. However, their impact on the volume distribution increases with larger diameters. In order to avoid an overrepresentation of these larger particles, two particles (*d_TEM_* = 430 Å and *d_TEM_* = 1040 Å) were excluded from the distribution of *c* = 0.1 mol L^−1^. The SAXS distribution does not show indications of particles larger than 200 Å, since this marks the end *q_min_* of the calculated spectrum for the SAXS distributions.

Nonetheless, these distributions are in good agreement with the findings shown so far. Although the primary particles are very polydisperse, with single large particles up to 1000 Å, the average diameter below 100 Å is quite small. This suggests the gas-to-particle route as the particle formation mechanism. In this route, the particles are formed of the vaporous precursor and typically in the low nanometer area. All other mechanisms mentioned in the introduction predict particles or shell of particles in the submicron and micron range. Because of the absence of at least submicron particles from SAXS and TEM data, a combination of mechanisms can be excluded as well.

### 3.3. Fractal Properties

**Figure 8:**
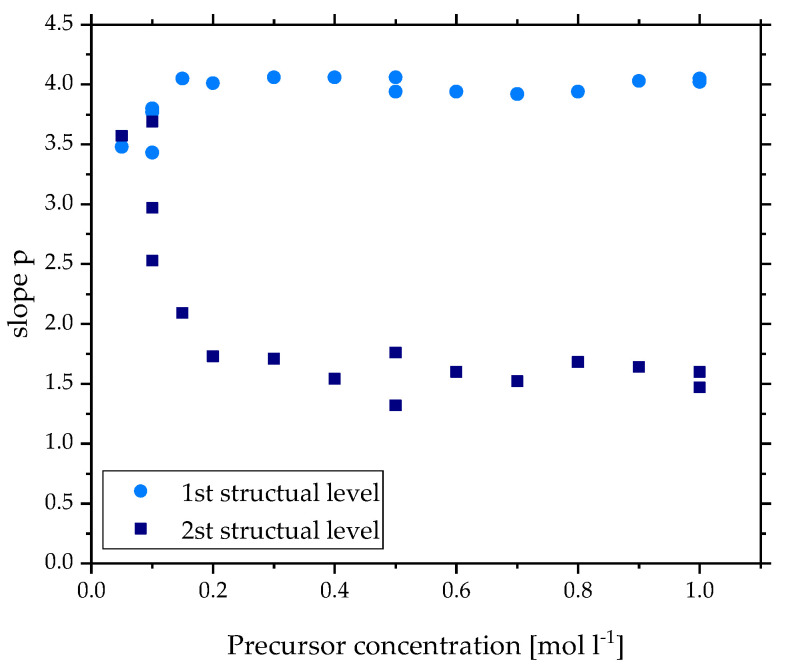
Slope *p* of the power law fit from SAXS scattering data for both structural levels of zirconia made by SFS with different precursor concentrations.

Another way to confirm the gas-to-particle formation mechanism is the analysis of the fractal properties. This allows the definition of the aggregates’ shape and could exclude spherical particles in the submicron range. In SAXS theory, the fractal dimension describes the fractal properties of the particle system with the slope or exponent *p* of the power law fit (Equation (3)). For the discussed aggregates of zirconia, it was possible to fit the 1st and 2nd structural level, resulting in two different types of fractal dimension: one to describe the surface of the primary particles in the 1st level and one to describe the shape of the aggregate in the 2nd level. Figure 8 shows the slope *p* for all precursor concentrations for the 1st (light blue) and 2nd (dark blue) structural level. Starting with the surface of the primary particles, the slope *p* lies around the value four for *c* > 0.2 mol L^−1^, showing typical Porod behavior. The particles have a sharp interface, resulting in smooth surfaces. The TEM images in Figure 4a confirm this property. For *c* < 0.2 mol L^−1^, *p* reaches values below 3.5 describing rough surfaces. Unfortunately, the resolution of the TEM images is not high enough to show the surface of these small particles in detail. However, this behavior has been reported for primary particles of carbon black as well [39]. In the case of zirconia, one reason for the change of the surface property could be the size. As described before, the primary particles of the lowest concentrations are the smallest and assume diameters under 50 Å. Because of the fast synthesis in the flame, these particles are formed quickly without time to perfect their shape. The principle “The smaller the particle the greater the surface to volume ratio” could explain why these imperfections of the particle have a stronger impact on smaller particles.

In the 2nd structural level, the slope *p* describes the shape of the aggregates with the fractal dimension of mass. Here, the aggregates of *c* > 0.2 mol L^−1^ assume values for the slope *p* between 1.32 and 1.76. These values translate to shapes between the first and second dimensions resulting in flaky aggregates with stringy parts. This is also observed in the TEM images in Figure 4a, although the outline of one aggregate cannot be defined clearly, as the overlapping of different aggregates is a high possibility. These values are lower than typically expected for flame-made products, as explained in Section 2.2. However, Hyeon et al. derived fractal dimensions of 1.6 and 1.72 for flame-made silica using SAXS, demonstrating some deviation from this theory [17]. Nonetheless, these values for the mass fractals clearly confirm fractal aggregates, strengthening the claim of the gas-to-particle route as a formation mechanism.

For *c* < 0.2 mol L^−1^, the slope *p* reaches values as high as 3.7, exceeding the highest logical value of three for mass fractals. However, this is in the range for surface fractals indicating larger particles. As seen in the TEM image (Figure 4a), single larger particles were detected for *c* = 0.1 mol L^−1^. Because of their size, these few particles should also impact the scattering behavior but were not detected in the calculated volume distribution due to the limit of the fitting range. In this case, the surface fractals of the larger particles and the mass fractals of the smaller particles are overlapping in the same scattering vector range. This leads to a value between two and four for the slope *p* depending on the number of large particles. Additionally, it effects the region of the Guinier fit, leading to varying *R_g_*_1_ for *c* = 0.1 mol L^−1^ in Figure 2a. Since the size of these larger particles varies greatly and numbers are low, it is unsurprising that no clear Guinier region could be detected for these particles. However, there are no indications in the TEM images that the size of these particles exceeds 5000 Å, indicating the gas-to-particle mechanism also for low concentrations. 

## 4. Conclusions

This study set out to investigate the influence of the precursor concentration on the fractal particle structure of zirconia using the standardized *spraysyn* burner for SFS to draw conclusions about the underlying particle formation mechanisms. The characterization of the metal oxide nanoparticles was carried out using SAXS and TEM with a focus on primary particles, their size distributions and fractal properties of the particle system. All these different parameters in combination with the conclusive results of both measurement techniques lead to such a detailed description of the fractal structures that the gas-to-particle route could be identified as the particle formation mechanism by excluding others.

In detail, low concentrations (*c* < 0.2 mol L^−1^) show inconclusive results and point to unstable process conditions during the fast zirconia formation in the flame. Both TEM and SAXS depict the smallest and largest primary particle sizes with a very high polydispersity, resulting in a low reproducibility. However, only particles smaller than half a micron were detected.

For medium (*c* = 0.2–0.6 mol L^−1^) and high precursor concentrations (*c* = 0.7–1.0 mol L^−1^), both measurement techniques are in good agreement about a stable primary particle size, indicating a stable and reproducible process. The averaged diameter for both concentration regimes is approximately 50% larger than for low concentrations, showing no further signs of growth. Although the polydispersity is quite high, it ranges around the self-preserving limit for aerosol growth for the free-molecular regime of particle transport. Volume distributions calculated from SAXS data reveal multimodal behavior for the primary particles. TEM is in good agreement with the 1st peak of the distribution but shows only a few outliners instead of further peaks. This characteristic illustrates that only a few larger particles can lead to a high polydispersity. Fractal properties show smooth surfaces for the primary particles. The fractal dimension is slightly lower than expected for diffusion-limited cluster aggregation but not out of range for flame-made products.

Together, these results lead to a detailed description of fractal aggregates produced with different precursor concentrations using SFS. This fractal structure and the absence of particles larger than half a micron point to the gas-to-particle route as a particle formation mechanism.

## Figures and Tables

**Table 1:** Range of scattering vectors for the size distribution fit as well as the slope *p*_1_ of the 1st power law fit.

Precursor Concentration *c* [mol L^−1^]	*q_min_* [Å^−1^]	*q_max_* [Å^−1^]	Slope *p*_1_
0.1	0.0353	0.108	3.5
0.4	0.0152	0.077	4.0
0.8	0.0152	0.077	4.0

**Table 2:** Diameters of the peak positions of the volume size distribution calculated from SAXS data of Figure 6.

Precursor Concentration *c* [mol L^−1^]	1st Peak [Å]	2nd Peak [Å]	3rd Peak [Å]
0.1	55	115	-
0.4	80	160	235
0.8	80	160	235

## Data Availability

The data presented in this study are available on request.

## Supplementary Materials

The following supporting information can be downloaded at: https://www.mdpi.com/article/10.3390/ma15062124/s1. Figure S1: WAXS scattering data for zirconia made by SFS with *c* = 0.4 mol L^−1^ and reference data for cubic and tetragonal crystal structure of zirconia (calculated X-ray diffraction patterns).
