# Supplementary material for: Characterization of Fractal Structures by Spray Flame Synthesis Using X-ray Scattering
Source: Materials (Basel). 2022 Mar 14;15(6):2124. doi: 10.3390/ma15062124 (PMC8950271; doi:10.3390/ma15062124)
Supplement: Supplementary file 1 [file materials-15-02124-s001.zip › materials-1586726-supplementary.pdf]

materials

Supplementary Material

Characterization of Fractal Structures by Spray Flame Synthesis Using X-ray Scattering

Mira Simmler *, Manuel Meier and Hermann Nirschl

Institute of Mechanical Process Engineering and Mechanics, Karlsruhe Institute of Technology, 76131 Karlsruhe, Germany; manuel-meier@outlook.com (M.M.); hermann.nirschl@kit.edu (H.N.)
* Correspondence: mira.simmler@kit.edu; Tel.: +49-721-608-44141

Supplementary Material

To demonstrate that zirconia was formed during the spray flame synthesis (SFS), Figure S1 shows WAXS (wide angle X-ray scattering) data of zirconia produced with a precursor concentration $c = 0.4$ mol l^{-1} of zirconium butoxide in butanol (blue) and references for cubic (red) and tetragonal (gray) crystal structure in the range of $20° < 2\theta < 55°$. The pattern displays a mixture of both phases with a higher proportion of the cubic phase.

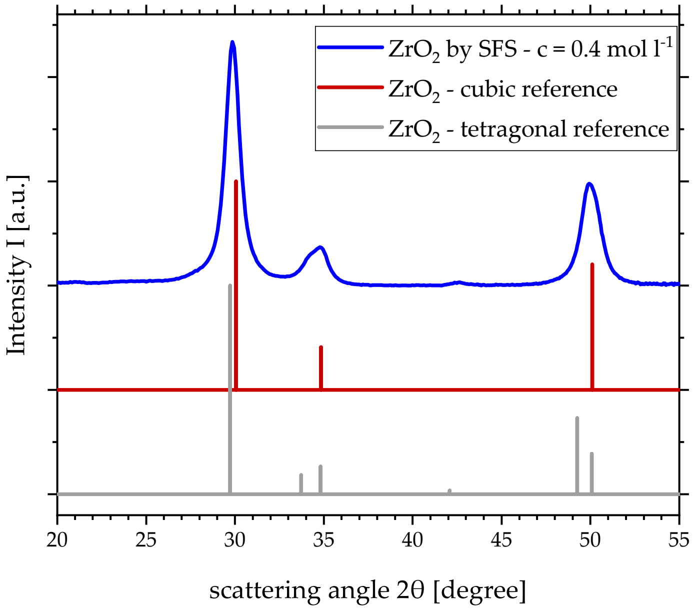

Figure S1: WAXS scattering data for zirconia made by SFS with $c = 0.4$ mol l^{-1} and reference data for cubic and tetragonal crystal structure of zirconia (calculated X-ray diffraction patterns) [26-28]

References

26. Persson, K. Materials Data on ZrO2 tetragonal. mp-2574. *The Materials Project*: Berkeley, CA, USA, **2014**. https://doi.org/10.17188/1200956. Available online: https://materialsproject.org/materials/mp-2574/ (accessed on 20 January 2022).
27. Persson, K. Materials Data on ZrO2 cubic. mp-1565. *The Materials Project*: Berkeley, CA, USA, **2014**. https://doi.org/10.17188/1191294. Available online: https://materialsproject.org/materials/mp-1565/ (accessed on 20 January 2022).
28. Jain, A.; Ong, S.P.; Hautier, G.; Chen, W.; Richards, W.D.; Dacek, S.; Cholia, S.; Gunter, D.; Skinner, D.; Ceder, G.; et al. Commentary: The Materials Project: A materials genome approach to accelerating materials innovation. *APL Materials* **2013**, 1, 11002, doi:10.1063/1.4812323.
